# Comparative analysis of the venom proteome of four important Malaysian snake species

**DOI:** 10.1186/1678-9199-20-6

**Published:** 2014-03-04

**Authors:** Jaya Vejayan, Too Lay Khoon, Halijah Ibrahim

**Affiliations:** 1 Faculty of Industrial Sciences and Technology, University Malaysia Pahang, Lebuhraya Tun Razak, Kuantan, Pahang Darul Makmur 26300, Malaysia; 2 Institute of Biological Sciences, University of Malaya, Kuala Lumpur 50603, Malaysia

**Keywords:** Mass spectrometry, Snake venoms, Proteomics, Two-dimensional gel electrophoresis

## Abstract

**Background:**

*Naja kaouthia*, *Ophiophagus hannah*, *Bungarus fasciatus* and *Calloselasma rhodostoma* are four venomous snakes indigenous to Malaysia. In the present study, their proteomic profile by two-dimensional gel electrophoresis (2-DE) have been separated and compared.

**Results:**

The 2-DE of venoms of the four species snake demonstrated complexity and obvious interspecies differences in proteome profiles. A total of 63 proteins were identified in the four species: *C. rhodostoma* – 26, *N. kaouthia* – 16, *O. hannah* – 15 and *B. fasciatus* – 6.

**Conclusions:**

Despite the identifications of major proteins in the four snake species, a large number of protein spots from the 2-DE were unidentified even though the spots displayed high-quality MALDI-TOF-MS spectra. Those identified included phospholipase A_2_ proteins in all four venoms, long neurotoxins in both cobra species and the *C. rhodostoma* venom found with the most varied types of peptidases, i.e. metalloproteinase kistomin, halystase and L-amino acid oxidase.

## Background

In Malaysia, more specifically Peninsular Malaysia, a very rich snake fauna is present, consisting of approximately 141 known species of land and sea snakes. Of these, only 16 species of land snakes and all 21 sea snake species are venomous [1]. This study focused on four of these venomous species, namely *Naja kaouthia*, *Ophiophagus hannah, Bungarus fasciatus* and *Calloselasma rhodostoma*. The first three are of the Elapidae family while *C. rhodostoma* belongs to the Viperidae family. The venoms of *N. kaouthia* (monocled cobra, 1.5-2.0 meters long), *O. hannah* (king cobra, 3 to 4 meters long) and *B. fasciatus* (banded kraits, 1.6 meters long) are comprised mainly of neurotoxins [2,3]. Other potent basic polypeptides – such as cardiotoxin, cytotoxin and cobramines – are also found abundantly in the venoms of elapids. The short-tempered, quick-to-attack cobra is one of the most frightening; while kraits, though much more subdued, are also highly feared for their toxic, frequently death-causing bites. The venom of *C. rhodostoma* (Malayan pit viper, 0.6-1 meters long, previously known as *Agkistrodon rhodosto*ma and *Ancistrodon rhodostoma*), is rich in peptidases that exhibit hemorrhagic activities and is capable of affecting blood coagulation, leading to a hemotoxic effect [4].

Since the early biochemical characterization of venoms, scientists have been unravelling the biological and pathological significance of their proteins and peptides. The advances in mass spectrometry (MS) for protein identification have revolutionized snake venom proteomics, shedding light into the global complexity of venom proteomes and, subsequently, their pathological activities. Using a multitude of proteomic approaches, venom proteomes have been analyzed in 55 snake genera [3]. The application of proteomics, including the use of two-dimensional gel electrophoresis (2-DE) and MS in studying venoms, has attracted considerable attention from researchers. The effective use of the 2-DE technique, coupled with MS, was first demonstrated in a study conducted by Fox and colleagues [5] in their comparative analysis of the venoms of *Dispholidus typus*, *Crotalus atrox* and *Bothrops jararaca*. The correlation between genetic, ecological and phylogeny factors with intraspecific variations in the composition of the *Trimeresurus stejnegeri* venom was investigated by Creer and colleagues [6] by employing matrix-assisted laser desorption ionization-time-of-flight (MALDI-TOF)/MS and isoelectric-focusing (IEF) technologies. To further assess the extreme complexity of natural venoms, Li et al. [2] assessed the global venom proteomics profiles of *Naja naja atra* and *Agkistrodon halys* by a combination of four different approaches. Nawarak et al. [7], on the other hand, used 2-DE and MALDI-TOF MS to identify moderate- to high-molecular-mass glycoproteins in *N. kaouthia* venom, which had been previously fractionated by binding with concanavalin A. These approaches have also been utilized for the characterization of novel proteins that are yet to be inserted into protein databases. For instance, MALDI-TOF MS has been employed to determine the molecular mass of purified proteins while 2-DE has been used to ascertain both the molecular weights as well as pI values of isolated proteins [8-10].

Numerous additional investigations into venom proteomes and subproteomes, using a wide array of proteomic strategies, have provided novel insights into venom contents, their biological activities and the evolutionary relationships among snakes [3]. Nevertheless, as experienced by Li et al. [2], only 50% of the spots were confirmed to be venom proteins although approximately 80% of the gel spots from 2-DE displayed high-quality MALDI-TOF MS spectra. Scarcity of venom sequence databases for the analysis of MS data has posed a challenge to all snake venom proteomic studies. Proteomics tools provide enormous versatility in diverse applications, ranging from unravelling the complexity of varies venoms to potentially identifying the minute differences between very closely related organisms [11]. The current study aims to further underline the importance and challenges of proteomics in the study of snake venom by profiling the venom of four snake species indigenous to Malaysia.

## Methods

### Snake venoms

All venoms used were from common venomous snakes in Malaysia, obtained from a local vendor, Bukit Bintang Enterprise Sdn Bhd. The venoms were freeze-dried and stored at –20°C.

### Protein content determination

The protein content in the four venoms was estimated using the dye-binding technique of Bradford [12] with bovine serum albumin (BSA) at 2.0 mg/mL concentration, purchased from Thermo Scientific.

### Two-dimensional Gel electrophoresis (2-DE)

Eighteen-centimeter IPG strips (GE Healthcare, Sweden) with a linear pH range of 3 to 10 were rehydrated overnight with 340 μL of rehydration solution. After rehydration, the IPG strips were introduced with the venomous proteins (100 μg for silver staining and 300 μg for Coomassie blue staining) via a sample-loading cup. Prior to this the venomous proteins had been dissolved in 100 μL of rehydration solution containing 8 M urea, 2% (w/v) CHAPS, 20 mM DTT (dithiothreitol), 0.5% (v/v) IPG buffer, 0.002% (w/v) Bromophenol blue. Electrofocusing was carried out at 30 kVh using IPGphor (GE Healthcare) at 20°C according to the manufacturer’s instruction. Before the second dimensional electrophoresis, the IPG strips were equilibrated by two equilibration steps: reduction buffer with 50 mM Tris/HCL, pH 8.8, 6 M urea, 30% (v/v) glycerol, 2% (w/v) SDS, a trace of Bromophenol blue and 1% (w/v) DTT on a rocking table for ten minutes; alkylation buffer with 50 mM Tris/HCL, pH 8.8, 6 M urea, 30% (v/v) glycerol, 2% (w/v) SDS, a trace of Bromophenol blue and 2.5% (w/v) iodoacetamide for an additional ten minutes. The equilibrated strips were loaded and run on 15% polyacrylamide Laemmli gels (26 cm × 20 cm) using the Ettan Dalt II system (GE Healthcare) with a programmable power control, initially 0.5 W per gel for 40 minutes, followed by 15 W per gel till the dye front reached the bottom of the gel. The separated gel proteins were visualized by Coomassie brilliant blue.

### MALDI-TOF MS

The Coomassie-stained gels were scanned using the Image Scanner (Amersham Biosciences Limited, Sweden) and the spots were detected, edited and annotated with ImageMaster 2D Platinum® software (Amersham Biosciences Limited, Sweden). The stained spots were selected, excised and dehydrated with 50 μL of acetonitrile for 15 minutes. To rehydrate, the supernatant was then replaced with 25 μL of 25 mM NH_4_HCO_3_ for ten minutes. These steps of rehydration followed by dehydration were repeated to give a total of three washes. Finally, the spots were dried for five minutes using a centrifugal evaporator (SpeedVac®, Thermo Scientific, USA).

### In-Gel tryptic digestion

The dried spots were then re-suspended in 10 μL of 10 ng/μL trypsin in 25 mM NH_4_HCO_3_ and incubated for complete digestion at 37°C overnight. On completion, the gel was once again dehydrated with the addition of acetonitrile. Finally, the gel piece was removed and the supernatant was dried down in the SpeedVac® for 20 minutes.

### Sample preparation for MS

An equal amount of tryptic-digested samples was mixed together with a matrix solution consisting of α-cyano-4-hydroxy cinnamic acid in acetonitrile acidified with trifluroacetic acid (1 mg/mL). Thereafter, 0.4 μL of this mixture was spotted on the slide and air-dried.

### Mass spectrometry

MALDI-TOF mass spectra of peptide mixture were obtained on an Ettan MALDI-TOF-Pro mass spectrometer (Amersham Biosciences AB, Sweden) by a delayed ion source with nitrogen laser of 337 nm. The acceleration voltage was set to 20 kV with positive ion reflectron mode. The low mass rejection was activated. The instrument was calibrated externally with peptide samples of adrenocorticotropic hormone and angiotensin III that gave respective spectrum peaks at 897.531 and 2465.199.

### Database searching

Proteins were identified via monoisotopic masses (as shown in the table found in Additional file 1) with the aid of the Internet search program MASCOT (http://www.matrixscience.com) as well as ExPASy (http://www.expasy.org). For the MASCOT search, the search parameters were: NCBInr database, SwissProt and/or MSDB; species: Chordata vertebrates and relatives; missed cleavages: 1; enzyme: trypsin, tolerance: 1.0 Da and charge state: MH+. The parameters for the ExPASy search were: Swiss-Prot database; species: other vertebrata; missed cleavages: 1; enzyme: trypsin, ion mode: MH^+^.

### Ethics statement

This study required no approval by any animal ethics committee as decided by the Animal Ethics Committee of University Malaya based on reasoning that the study dealt with venom milked from snakes of the wild rather than experimenting on them in the laboratory. All precautions were taken to ensure no harm were inflicted on the snakes by an expert snake handler.

## Results

### Comparative analysis of 2-DE on various snake venoms

**Figure 1:**
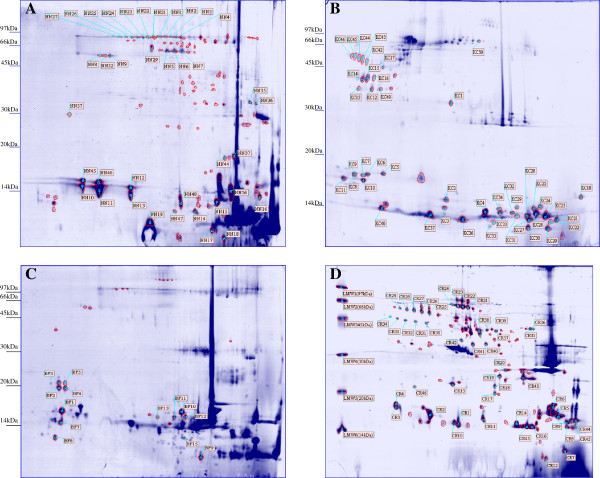
**Proteome maps of the snake venoms of (A) *N. kaouthia*, (B) *O. hannah*, (C) *B. fasciatus*, and (D) *C. rhodostoma *with IPG 3-10 (300 μg protein, 18 cm), 15% T, Coomassie blue staining showing the annotated spots of the four venoms.** The spots were detected and annotated by using the software Image Master 2D Platinum. These protein spots were identified from the 2-DE gel stained by Coomassie blue, tryptic-digested, extracted and analyzed in duplicates per spot with MALDI-TOF MS in reflectron mode.

In the first-dimension isoelectric focusing, 18 cm IPG (pH 3-10) strips were used with the cup-loading technique, which was found to produce better results than sample application by in-gel rehydration. It was also ideal for separating the more basic proteins of the snake venoms when the loading cup is applied at the anodic site. Subsequently, the proteins were separated in the second dimension by 15% SDS-PAGE. The 2-DE images of the four snake venoms demonstrated different degrees of complexity in polypeptide constituents in general. The visual inspection of 2-DE maps showed notable differences in proteome profiles among the venoms of *N. kaouthia, O. hannah, B. fasciatus* and *C. rhodostoma* (Figure 1). In particular, basic (high pI) and low-molecular-mass protein spots were found to be highly prominent in the venoms of *N. kaouthia* and *B. fasciatus*. This is typical of venom of Elapidae origin, which is comprised mainly of neurotoxins. Nawarak et al. [13] had also demonstrated similar proteomic profiles in snake venoms from Elapidae families using multidimensional chromatographic approaches. Nevertheless, the proteomic profiles of *O. hannah* displayed certain degrees of disparity against those of elapids and showed similarities with those of *C. rhodostoma*, where the venom proteins were evenly dispersed across the pI and mass range.

### Peptide mass fingerprinting (PMF) identification of protein spots from four snake venoms

Spots were selected and carefully identified from the 2-DE gel stained by Coomassie blue, tryptic-digested, extracted and analyzed in duplicates per spot with MALDI-TOF MS in reflectron mode. Identification by PMF on proteins isolated from 2-DE gels of the four venoms was performed by scanning the monoisotopic masses with two separate Internet search programs, MASCOT (http://www.matrixscience.com/cgi/search_form.pl?FORMVER=2&SEARCH=PMF) and ExPASy (previously located URL: http://www.expasy.org/tools/aldente/). In PMF, the profile of the unknown protein was compared with theoretical peptide libraries generated from sequences in the different databases. Identified proteins were chosen from either MASCOT or ExPASy, which yielded more numerous hits and higher sequence coverage. The latter search program was taken out of the public domain and continued as a licensed database until recently discontinued altogether.

A total of 113, 50, 26, and 115 spots were analyzed to obtain the venom protein compositions of *N. kaouthia, O. hannah, B. fasciatus,* and *C. rhodostoma*, respectively, but only the corresponding 34%, 86%, 54%, and 40% of these spots produced quality MALDI-TOF MS spectra. These spots were annotated and displayed in Figure 1. The remaining spots failed to give any spectra (not annotated within the figure). Respective totals of 16, 15, 6, and 26 spots linked to the database of *N. kaouthia*, *O. hannah, B. fasciatus*, and *C. rhodostoma* were identified by PMF (Table 1). Some proteins were mapped to more than one spot, for example, those of the cobra venom factor, phospholipase A_2_ isozymes and L-amino-acid oxidase. Although they produced relatively good quality spectra, a few spots from the 2-DE of the four venom species were unmatchable with proteins belonging to their respective venoms by either of the two database searches.

**Table 1:** **Protein identification of annotated spots in venom proteomes of *N. kaouthia*, *O. hannah*, *B. fasciatus *** **and *C. rhodostoma***

**Annotated spot**	**Database**	**Identified protein**	**Accession number**	**Hits**	**SC,%**	**Mw, kDa**	**pI**
***N. kaouthia***							
NN5	ExPASy	Hemorrhagic metalloproteinase kaouthiagin	P82942	6	13	44493	6.66
NN8	ExPASy	Cobra venom factor beta chain	Q91132	7	20	43574	5.26
NN9	MASCOT	Cobra venom factor precursor	AAA68989	10	6	184401	5.99
NN10	ExPASy	Phospholipase A_2_ isozyme 2	P00597	4	39	13271	5.16
NN11	ExPASy	Phospholipase A_2_ isozyme 2	P00597	7	67	13271	5.16
NN12	ExPASy	Phospholipase A_2_ isozyme 1	P00596	3	35	13456	4.91
NN13	ExPASy	Phospholipase A_2_ isozyme 2	P00597	4	41	13271	5.16
NN14	ExPASy	Long neurotoxin 1 (neurotoxin 3) (alpha-cobratoxin)	P01391	2	34	7831	8.59
NN15	ExPASy	Muscarinic toxin-like protein 2 (MTLP-2)	P82463	8	85	7299	8.18
NN16	ExPASy	Thaicobrin	P82885	6	77	12038	9.16
NN17	ExPASy	Muscarinic toxin-like protein 3 (MTLP-3)	P82464	2	45	7624	8.13
NN19	ExPASy	Cytotoxin 1	P60305	2	28	6701	9.16
NN37	MASCOT	Thaicobrin	P82885	4	44	12030	9.17
NN45	ExPASy	Phospholipase A_2_ isozyme 1	P00596	4	39	13456	4.91
NN46	ExPASy	Phospholipase A_2_ isozyme 1	P00596	2	17	13456	4.91
NN52	ExPASy	Cobra venom factor beta chain	Q91132	4	11	43574	5.26
***O. hannah***							
KC5	ExPASy	Phospholipase A_2_	Q9DF56	2	22	13931	4.83
KC6	ExPASy	Phospholipase A_2_, acidic 2	Q9DF33	4	33	13191	4.41
KC9	ExPASy	Phospholipase A_2_, acidic 2	Q9DF33	4	44	13191	4.41
KC10	ExPASy	Phospholipase A_2_, acidic 2	Q9DF33	3	19	13190	4.42
KC18	ExPASy	Ohanin	P83234	5	57	11952	9.57
KC20	ExPASy	Trypsin and chymotrypsim bi-functional serine protease inhibitor	B6RLX2	5	86	6346	8.59
KC23	ExPASy	Long neurotoxin 1 (neurotoxin A)	P01387	2	47	8106	8.04
KC24	MASCOT	Thioredoxin protein	Q98TX1	3	38	11995	5.30
KC25	ExPASy	Long neurotoxin OH-17	Q53B54	4	50	8037	8.34
KC26	ExPASy	Long neurotoxin OH-55	Q53B58	7	93	7919	8.34
KC27	ExPASy	Long neurotoxin 4	P80156	3	36	8014	8.05
KC32	ExPASy	Muscarinic toxin-like protein 3 homolog	A8N286	2	22	7542	7.44
KC33	ExPASy	Long neurotoxin OH-55	Q53B58	2	42	7919	8.34
KC37	ExPASy	Weak toxin DE-1	P01412	2	32	7027	5.49
KC50	ExPASy	Complement-depleting factor	ABN72543	9	6	184237	6.12
***B. fasciatus***							
BF1	ExPASy	Phospholipase A_2_ homolog	P29601	3	39	13104	4.37
BF2	ExPASy	Phospholipase A_2_ KBf-grIB	P0C551	3	26	14157	4.78
BF4	ExPASy	Phospholipase A_2_ KBf-grIB	P0C551	3	26	14157	4.78
BF7	ExPASy	Phospholipase A_2_ homolog	P29601	2	19	13104	4.37
BF9	ExPASy	Phospholipase A_2_ 13	P00627	3	71	3704	8.91
BF15	ExPASy	Phospholipase A_2_ isozyme 6	P00627	2	25	13066	7.94
***C. rhodostoma***							
CR1	ExPASy	Phospholipase A_2_ H1E6	Q9PVF2	8	48	13919	5.51
CR2	ExPASy	Rhodocetin alpha subunit	P81397	8	67	15962	5.16
CR5	ExPASy	Phospholipase A_2_ W6D49	Q9PVF4	10	40	13689	8.34
CR6	ExPASy	Phospholipase A_2_ W6D49	Q9PVF4	11	40	13689	8.34
CR14	ExPASy	Disintegrin rhodostomin	P30403	8	93	7330	6.70
CR15	ExPASy	Rhodocytin subunit beta	Q9I840	6	69	14369	7.08
CR16	ExPASy	Rhodocytin subunit beta	Q9I840	6	69	14369	7.08
CR17	ExPASy	Metalloproteinase kistomin	P0CB14	2	9	25846	6.41
CR18	ExPASy	Metalloproteinase kistomin	P0CB14	2	9	25846	6.41
CR19	ExPASy	Halystase	P81176	2	14	26483	6.70
CR21	MASCOT	L-amino acid oxidase	AJ271725	7	22	58184	6.05
CR22	MASCOT	L-amino acid oxidase	AJ271725	15	31	58184	6.05
CR23	ExPASy	L-amino acid oxidase	P81382	12	26	56228	6.04
CR25	ExPASy	Ancrod (EC 3.4.21.74) (venombin A) (protein C activator)	P26324	7	27	26571	8.59
CR27	ExPASy	Ancrod (EC 3.4.21.74) (venombin A) (protein C activator)	P26324	9	27	26571	8.59
CR28	MASCOT	Venombin A (EC 3.4.21.74)	S20407	5	16	26553	8.61
		1 [validated]					
CR29	MASCOT	Venombin A (EC 3.4.21.74)	S20407	4	16	26553	8.61
		1 [validated]					
CR30	MASCOT	Ancrod-like protein	CAA01524	3	10	25213	8.44
CR31	MASCOT	Ancrod-like protein	CAA01524	4	14	25213	8.44
CR32	MASCOT	Ancrod-like protein	CAA01524	4	14	25213	8.44
CR33	MASCOT	Ancrod-like protein	CAA01524	3	10	25213	8.44
CR34	MASCOT	Ancrod-like protein	CAA01524	14	4	25213	8.44
CR39	MASCOT	L-amino acid oxidase	AJ271725	8	21	58184	6.05
CR40	MASCOT	Ancrod-like protein	CAA01524	4	13	29314	8.54
CR41	ExPASy	Ancrod [precursor]	P47797	4	13	26461	8.53
CR43	ExPASy	Metalloproteinase kistomin	P0CB14	2	9	25846	6.41

## Discussion

In this study, the 2-DE of the various snake venoms displayed prominent vertical bands that hampered the identification of bioactive proteins. Similar bands were also observed by other researchers. For instance, Marshall and Williams [14] as well as Saravia et al. [15] reported similar findings in their works on *Crotalus* and *Bothrops asper* snake venoms, respectively.

As expected, the 2-DE images from the venoms of the four snake species demonstrated the complexity of polypeptide constituents and the known fact that the composition of snake venoms is 70 to 90% proteins [16]. The 2-DE of the four species showed obvious variations in protein map patterns. The distinctive protein maps obtained in this study may enable species identification, which is of clinical importance. The characteristic protein map of *O. hannah*, which displayed dissimilarities in relation to the other two elapids, exemplified the significance of this purpose. The 2-DE venom proteome profile of *O. hannah* displayed numerous protein spots at the higher molecular mass region similar to the viper rather than the other two elapids. This finding is substantiated by reports from Ohsaka [17] describing the exceptional existence of hemorrhagic activity provoked by *O. hannah* venom similar to that exerted by Viperid venoms while none of the Elapidae venoms exhibits similar hemorrhagic activity. The hemorrhagic activity of the major hemorrhagin, known as hannah toxin in *O. hannah*, was first demonstrated by Tan and Saifuddin [18] in rabbits. Further investigation may enable potential species-specific molecular markers to be identified or detected under different gel conditions, similar to biomarkers for the purpose of species identification or disease specification (e.g. in prognosis/diagnosis of various cancers). A study by Guércio et al. [19] had also postulated the potential of differential analyses of snake venom proteomes on the development of ontogenetic molecular markers via the identification of group-specific proteins.

To assess the venoms’ proteomics profiles, the combination of 2-DE with MALDI-TOF was used. Our study, unlike that done by Li et al. [2], matched the venom peptide masses of the particular snake species to their corresponding protein databases, found in the Taxonomy Browser. The Taxonomy Browser contained in the Entrez database (http://www.ncbi.nlm.nih.gov) provided the total number of proteins available for matching for each of the four snake venoms. The respective numbers of known proteins available as of October 2013 were 157 for *N. kaouthia*, 192 for *O. hannah*, 130 for *B. fasciatus* and 84 for *C. rhodostoma*. The protein database was constructed based on the sequence data from the translated coding regions from DNA sequences in GenBank, EMBL, and DDBJ as well as protein sequences submitted to Protein Information Resource (PIR), SWISS-PROT, Protein Research Foundation (PRF), and Protein Data Bank (PDB) (sequences from solved structures). It was obvious that the database was incomplete for snake venom matching, compared with 784,406 proteins available for *Homo sapiens* (human), and 123,173 for *Bos taurus* (cattle) proteins. Nevertheless, the snake venom protein database is still of greater value than those of some other venomous species such as *Chironex yamaguchii* (sea wasp) with only two proteins, *Hadrurus aztecus* (scorpion) with one, and *Dolomedes plantarius* (spider) with none, to name a few, for matching.

A closer examination of the snake-venom-protein database disclosed the following information: some of the proteins were not found in the venom but were evidently found elsewhere in the snake’s body, for example, the nerve growth factor (located in the nervous system), NADH dehydrogenase and cytochrome b (located in mitochondria) and oocyte maturation factor (located in the reproductive system). Also, most of the venom proteins were precursor forms or polypeptide chains of the same protein, for example: phospholipase PLA_2_ precursor and chain A, crystal structure of L-amino acid oxidase. Based on these factors, therefore, the snake venom proteins available for matching in the database were, in fact, much more limited.

Nevertheless, even with these limitations, many important proteins were identified in the 2-DE of the 4 species. A total of 63 proteins were identified: *C. rhodostoma* - 26, *N. kaouthia* - 16, *O. hannah* – 15 and *B. fasciatus*– 6. Venoms from snakes contain various types of toxins such as cardiotoxins and neurotoxins, biological factors that are anti-coagulant, anti-thrombotic, anti-platelet binding, as well as active enzymes including phospholipase, endo- and exonucleotidases, hyaluronidase, neuraminidase, protease and many others. Generally, the non-enzymatic protein or polypeptide toxins dominate the lethal actions of elapids (e.g. cobra and krait) and sea snakes, whereas enzymes appear to play a more important role in the lethality of viperid (viper) and crotalid (pit viper) venoms [16].

In the present study, the phospholipase A_2_ (PLA_2_) group of enzymes was identified in all four species. At least one of these enzyme types was identified in each of the four species studied (Table 1). This finding is not surprising given that extensive studies on venom have demonstrated an accelerated natural selection force that drives the evolution of a multitude of extremely potent snake toxins from an ancestral PLA_2_ with digestive function [20,21]. Hundreds of species of venomous snakes of the families Elapidae and Viperidae were shown to have evolved a wide variety of venoms that contain varying proportions of toxins endowed with PLA_2_ activity, characterized by their neurotoxicity, myotoxicity, as well as anticoagulant and edema-inducing properties [21-23]. Hence, in snake venom, PLA_2_ enzymes, in addition to their possible role in digesting the prey, exhibit a wide variety of pharmacological effects through interfering with normal physiological processes [24]. It is well known that some of the most toxic and potent pharmacologically active components of snake venoms are either PLA_2_ enzymes or their protein complexes. For example, all known presynaptic neurotoxins from snake venom are PLA_2_ enzymes or contain at least one PLA_2_ as an integral part [25,26]. PLA_2_ myotoxins are more potent and act faster than their non-enzymatic counterparts [27].

PMF using MS also allowed the identification of long neurotoxins in both the cobra species studied. Unlike presynaptic beta-neurotoxins that exhibit varying PLA_2_ activities, these long neurotoxins are classified as alpha-neurotoxins that affect the post-synaptic membrane [28]. Post-synaptic neurotoxins have been identified only in venoms from the families Elapidae and Hydrophiidae (sea snakes). They are nicotinic receptor antagonists on the skeletal muscle and display different binding kinetics and affinity for subtypes of nicotinic receptors [29]. Other significant neurotoxins, such as the muscarinic toxin-like proteins (MTLP), including ohanin and thaicobrin, were also identified in the cobra venoms.

Rhodocetin, another important protein identified in *C. rhodostoma* venom, is a Ca^2+^-dependent lectin-related protein (CLP) that is a potent platelet aggregation inhibitor induced by collagen. It is a prime example of a CLP dimer, in which the two subunits are held together by non-covalent interactions and act synergistically to elicit the biological activity, affecting platelet aggregation and blood coagulation, important in cellular thrombosis and non-cellular processes in homeostasis [30,31]. This protein was also demonstrated to antagonize stromal tumor invasion *in vitro* and other α2β1 integrin-mediated cell functions [32]. The proteomic approach applied in the present study also successfully identified a significant number of peptidases, namely zinc metalloproteinase or disintegrin, ancrod or venombin A, and L-amino-acid oxidases which display the well-documented hemotoxic properties of the viper's venom. Disintegrin exhibits hemorrhagic activities by binding to the glycoprotein IIb-IIIa receptor on the platelet surface, thus inhibiting fibrinogen interaction with platelet receptors while L-amino-acid oxidases exert hemorrhagic effects by catalyzing oxidative deamination of hydrophobic and aromatic L-amino acids [33-36]. L-amino-acid oxidases have also recently been shown to display antibacterial properties [37]. On the other hand, ancrod is a thrombin-like serine protease that selectively cleaves the fibrinopeptides, resulting in aberrant fibrinogen that is unable to form dispersible blood clots [38].

Despite the identification of major proteins in the four snake species, a large number of spots from the 2-DE were unidentifiable with their respective snake venom protei ns even though the spots displayed high-quality MALDI-TOF-MS spectra. This was similar to the finding of another research group who identified proteins in two types of snake venoms: *Naja naja atra* and *Agkistrodon halys*[2]. In their study, only 50% of the spots were confirmed to have venom properties from approximately 80% of 2-DE gel spots displaying high-quality MALDI-TOF-MS spectra. As mentioned earlier, this was due to the unavailability of a full protein database or the result of post-translational modification (PTM). PTM of venomous proteins is a common phenomenon in snake venoms. Of a number of mechanisms inducing chemical modification of protein, glycosylation is one of the most frequently found Li et al. [2]. For instance, different degrees of glycosylation were discovered in the snake venom toxins of *Crotalus atrox* and *Bothrops jararaca*, using the fluorescent glycoprotein specific stain, Pro-Q-Emerald [39]. Given that the database for snake venoms is still developing, it is worth noting that easier identification remains possible in the future. This is evident when one compares the number of known proteins dating back to October 2005 (67 for *N. kaouthi*a, 78 for *O. hannah*, 34 for *B. fasciatus* and 60 for *C. rhodostoma*) to the most recent Entrez protein database. Among these the protein database of *B. fasciatus* shows the most promising development, an augmentation of approximately 380%, while those of *O. hannah* increased by 125%, *N. kaouthia* by 235%, and *C. rhodostoma* by 140%.

With the comprehensive cataloguing of venom toxins, the characterization of species-specific venom proteome is therefore greatly facilitated when studying the patho-physiological effects arising from species-specific snakebites. For instance, the sequential, comparative 2-DE analysis of *in vivo* and *in vitro* effects of venom on specific plasma proteins from a patient bitten by a rattlesnake has demonstrated the pathological relationship between the snake toxins and the injury-related physiological alterations [40]. The application of proteomic tools by Escalante et al. [41] on wound exudates also revealed the tissue-damaging mechanisms of different snake venom toxins.

## Conclusions

The 2-DE map of these venoms will play a vital role in the purification of their proteins by utilizing the 2-DE-guid purification applications as described by Tang et al. [42].

## Competing interests

The authors declare that they have no competing interests.

## Authors’ contributions

All authors contributed equally to the present work. All authors read and approved the final manuscript.

## Supplementary Material

Additional file 1 The peptide masses used for identifying the proteins. Click here for file
