# Supplementary material for: Comparative analysis of the venom proteome of four important Malaysian snake species
Source: J Venom Anim Toxins Incl Trop Dis. 2014 Mar 4;20:6. doi: 10.1186/1678-9199-20-6 (PMC4015498; doi:10.1186/1678-9199-20-6)
Supplement: Additional file 1 — The peptide masses used for identifying the proteins. [file 1678-9199-20-6-S1.docx]

| **Annotated spot** | **Peptide mass (m)** |
| --- | --- |
| NN5 | 826.652  880.552  981.508  1099.585  1274.619  1692.714 |
| NN8 | 991.601  1032.637  1176.802  1337.830  1444.979  1489.962  1648.928 |
| NN9 | 951.2590  1032.4550  1176.5430  1231.5630  1293.5030  1337.6120  1444.6610  1489.7150  1730.8070  1791.6860 |
| NN10 | 1157.566  1205.631  1697.689  1842.660 |
| NN11 | 812.459  1157.679  1188.675  1205.708  1610.861  1697.968  1842.996 |
| NN12 | 1697.968  1769.943  1842.995 |
| NN13 | 812.478  1610.859  1697.952  1769.923 |
| NN14 | 1315.668  1547.938 |
| NN15 | 995.619  1011.587  1151.703  1319.716  1576.922  1613.858  2188.212  2344.355 |
| NN16 | 913.551  1078.661  1495.925  1536.046  1811.139  2270.485 |
| NN17 | 1177.751  2510.584 |
| NN19 | 2091.359  2107.371 |
| NN37 | 913.8420  1079.0500  1536.5470  1811.5660 |
| NN45 | 900.507  1232.659  1842.764  2182.917 |
| NN46 | 900.587  1842.733 |
| NN52 | 1032.509  1176.639  1337.675  1648.712 |
| KC5 | 1249.631  2153.909 |
| KC6 | 1214.598  1230.562  1583.471  1817.425 |
| KC9 | 1583.778  1739.859  1817.821  2877.481 |
| KC10 | 1214.572  1230.585  1583.634 |
| KC18 | 913.500  930.549  1078.549  1497.644  2269.758 |
| KC20 | 944.586  1257.657  1414.728  1532.813  1994.823 |
| KC23 | 1210.458  2954.082 |
| KC24 | 1473.7080  1493.7840  1638.8510 |
| KC25 | 1205.776  1317.798  1361.903  1786.988 |
| KC26 | 981.578  1210.711  1571.733  1628.809  1756.958  2026.318  2954.435 |
| KC27 | 1301.706  1882.075  1898.099 |
| KC32 | 820.577  1076.628 |
| KC33 | 1210.769  2125.274 |
| KC37 | 1104.505  1300.601 |
| KC50 | 931.6520  952.4100  1136.7030  1316.9950  1386.9480  1498.1130  1715.1880  1910.4150  1970.2590 |
| BF1 | 1302.539  1670.597  2586.984 |
| BF2 | 955.532  1369.618  1746.644 |
| BF4 | 955.532  1726.642  1746.644 |
| BF7 | 1302.575  1670.625 |
| BF9 | 812.503  880.566  1051.556 |
| BF15 | 1708.712  1991.918 |
| CR1 | 826.468  980.545  1216.690  1372.934  1388.952  1505.767  1572.849  2024.183 |
| CR2 | 1175.618  1377.863  1432.823  1439.937  1843.094  1955.157  2254.355  2741.666 |
| CR5 | 824.405  925.468  941.461  1329.651  1457.773  1551.683  1707.766  1723.773  1739.805  1787.971 |
| CR6 | 925.528  941.544  1551.824  1567.803  1707.962  1723.944  1739.943  1817.154  1989.982  1997.131  2047.045 |
| CR14 | 814.569  1151.492  1187.570  1937.561  1953.581  2022.699  2345.623  2412.827 |
| CR15 | 1296.812  1568.990  2030.325  2087.324  2418.446  2545.541 |
| CR16 | 861.458  1296.539  2029.744  2086.660  2417.609  2544.590 |
| CR17 | 1032.624  1568.827 |
| CR18 | 1032.626  1412.781  1568.845  1672.764 |
| CR19 | 1323.006  2633.799 |
| CR21 | 1260.7500  1279.9950  1655.2460  2001.3390  2018.3780  2271.5720  3021.9570 |
| CR22 | 872.6680  877.2950  925.6440  1055.7240  1260.5850  1279.8460  1293.6910  1607.7190  1654.8540  2000.8200  2017.8830  2027.8580  2074.8450  2271.0720  3021.0340 |
| CR23 | 925.737  1260.676  1279.902  1293.757  1584.844  1607.787  1654.941  1712.911  1898.965  2017.957  2027.885  2271.114 |
| CR25 | 1175.700  1199.729  1260.755  1326.719  1378.792  1394.799  1512.799 |
| CR27 | 1104.670  1120.611  1175.718  1199.773  1260.734  1326.742  1378.796  1394.781  1512.815 |
| CR28 | 1019.8120  1104.8450  1175.8940  1199.9920  1379.1070 |
| CR29 | 1019.8070  1104.8120  1199.9920  1379.0980 |
| CR30 | 951.7120  1462.3940  1846.7680 |
| CR31 | 951.4780  1110.6830  1462.0890  1846.3760 |
| CR32 | 951.6170  1110.8550  1462.2250  1846.5150 |
| CR33 | 951.8150  1462.5370  1846.9180 |
| CR34 | 951.4620  1110.6440  1461.9390  1846.1320 |
| CR39 | 925.6990  1280.0840  1293.9460  1655.2760  2001.4120  2018.4270  2075.5540  3021.5270 |
| CR40 | 1132.9260  1217.0590  1311.1650  1390.2570 |
| CR41 | 1132.802  1216.816  1310.840  1389.949 |
| CR43 | 1032.734  1412.979 |
